# Upregulation of miR-572 transcriptionally suppresses SOCS1 and p21 and contributes to human ovarian cancer progression

**DOI:** 10.18632/oncotarget.3737

**Published:** 2015-03-30

**Authors:** Xin Zhang, Junling Liu, Dan Zang, Shu Wu, Aibin Liu, Jinrong Zhu, Geyan Wu, Jun Li, Lili Jiang

**Affiliations:** ^1^ Department of Pathophysiology, Guangzhou Medical University, Guangzhou, China; ^2^ Department of Experimental Research, State Key Laboratory of Oncology in Southern China, Sun Yat-sen University Cancer Centre, Guangzhou, China; ^3^ Department of Medical Oncology, State Key Laboratory of Oncology in Southern China, Sun Yat-sen University Cancer Center, Guangzhou, China; ^4^ Department of Biochemistry, Zhongshan School of Medicine, Sun Yat-sen University, Guangzhou, Guangdong, China

**Keywords:** ovarian cancer, miR-572, proliferation, SOCS1, p21

## Abstract

Ovarian cancer is a gynecological malignancy with high mortality rates worldwide and novel diagnostic and prognostic markers and therapeutic targets are urgently required. The suppressor of cytokine signaling 1 (SOCS1) and cyclin-dependent kinase inhibitor 1A (p21^KIP^) are known to regulate tumor cell proliferation. However, the mechanisms that regulate these genes have not yet been completely elucidated. In the present study, analysis of a published microarray-based high-throughput assessment (NCBI/E-MTAB-1067) and real-time PCR demonstrated that miR-572 was upregulated in human ovarian cancer tissues and cell lines. Kaplan-Meir analysis indicated that high level expression of miR-572 was associated with poorer overall survival. Ectopic miR-572 promoted ovarian cancer cell proliferation and cell cycle progression *in vitro* and tumorigenicity *in vivo*. *SOCS1* and *p21* were identified as direct targets of miR-572 and suppression of *SOCS1* or *p21* reversed the inhibiting-function of miR-572-silenced cell on proliferation and tumorigenicity in ovarian cancer cells. Additionally, the expression of miR-572 correlated inversely with the protein expression levels of SOCS1, p21 and positively with Cyclin D1 in ovarian carcinoma specimens. This study demonstrates that miR-572 post-transcriptionally regulates SOCS1 and p21 and may play an important role in ovarian cancer progression; miR-572 may represent a potential therapeutic target for ovarian cancer therapy.

## INTRODUCTION

Ovarian cancer is a gynecological malignancy with high mortality rates worldwide, and is the seventh most common cancer and the eighth most common cause of death due to cancer in females [1]. In 2012, 239,000 cases were diagnosed and 152,000 deaths due to ovarian cancer occurred worldwide [2]. The disproportionately high mortality rates and relatively poor prognosis of ovarian cancer can be attributed to the absence of clinical symptoms in early stage disease and the high failure rate for chemotherapy in advanced stage disease [3]. The five-year overall survival rate for early stage ovarian cancer is 70%–90% compared to only 20% for advanced stage disease [3]. In order to improve the survival of patients with ovarian cancer, it is important to identify novel prognostic markers and therapeutic targets [4-7].

Cancer is essentially a disease in which cells lose the normal checks on cell proliferation [8], and develops as a result of excessive cell proliferation. Therefore, exploration of the regulatory mechanisms that control tumor cell growth in order to target the cell proliferation machinery is an important strategy for cancer treatment [8, 9]. As a prototypic member of the suppressor of cytokine signaling (SOCS) family, SOCS1 was initially defined as an attenuator of the signaling of numerous cytokines via a feedback loop mechanism [10-12]. Altered SOCS1 expression has been reported in a wide range of human cancers and it may represent a diagnostic or prognostic biomarker. For example, *SOCS1* is frequently silenced by CpG island methylation in human hepatocellular carcinoma [13] and frequently inactivated by hypermethylation in multiple myeloma [14]. In human breast cancer, the mRNA expression of *SOCS1* decreases as TNM stage increases, and high *SOCS1* expression is significantly associated with earlier tumor stage and a better clinical outcome [15]. p21 plays essential roles in the cellular response to DNA damage, and functions as a regulator of cell cycle progression that its overexpression results in cell cycle arrest [16]. In a similar manner to SOCS1, p21 is expressed at higher levels in advanced stage head and neck cancer with longer overall survival [17]. Expression of p21 has also been reported to correlate inversely with T classification and clinical stage in squamous cell carcinoma of the tongue [18]. Although the expression of SOCS1 is lost in many human tumors and p21 is reported to be downregulated in multiple malignancies [19-22], the mechanisms leading to these effects are complicated and poorly understood.

MicroRNAs (miRNAs), small non-coding RNAs containing 20 - 22 nucleotides, are involved in various biological processes such as cellular differentiation, proliferation, oncogenesis and angiogenesis [23-26]. It has been demonstrated that miRNAs play important roles during malignant progression by negatively regulating their target mRNAs via recognizing and binding their 3` untranslated regions (3` UTRs) [27, 28]. The identification of the post-translational regulatory function of miRNAs has provided novel insight into tumor suppressor gene expression. As the expression of numerous miRNAs has been found to closely correlate with multiple biological aspects of cancer progression, miRNAs are considered to represent potential therapeutic targets for cancer [27-29].

Herein, we report that miR-572 is significantly upregulated in ovarian cancer and that the expression of miR-572 correlates with progression and overall survival in human ovarian cancer. Ectopic miR-572 promoted - while inhibition of miR-572 reduced - the proliferation, cell-cycle progression and tumorigenicity of ovarian cancer cells *in vitro*. Furthermore, we demonstrate that miR-572 directly targets and downregulates *SOCS1* and *p21* via recognizing their 3` UTRs, and downregulation of SOCS1 or p21 was essential for the miR-572-mediated effects in ovarian cancer cells. This study demonstrates that miR-572 plays an important role in the development and progression of ovarian cancer and may represent as a potential therapeutic target for ovarian cancer.

## RESULTS

### miR-572 is upregulated and correlates with overall survival in human ovarian cancer

**Figure 1:**
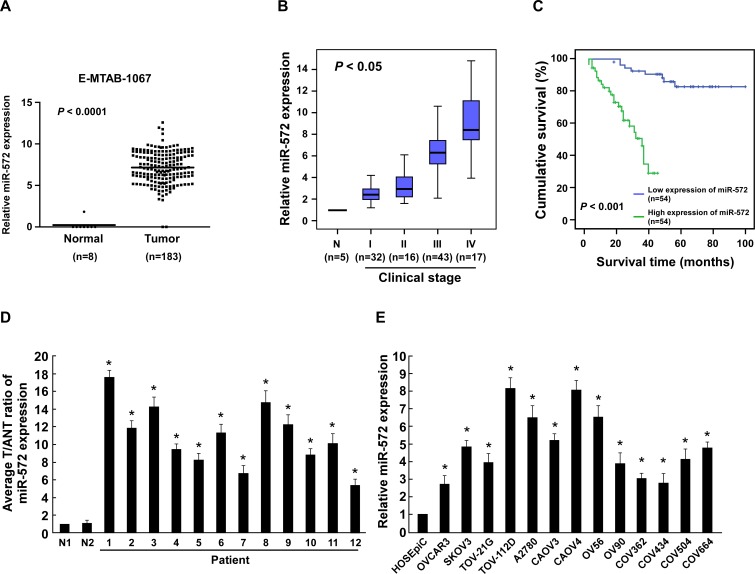
miR-572 is upregulated in ovarian cancer (**A**) Analysis of a public microarray data revealed that miRNA-572 was upregulated in human ovarian cancer tissues (*n* = 183) compared with normal ovarian tissues (*n* = 8; *P* < 0.0001; NCBI/E-MTAB-1067). (**B**) Real-time PCR analysis of miR-572 expression in different clinical stages of human ovarian cancer. Transcript levels were normalized to *U6* expression. The boundaries of the boxes represent the lower and upper quartiles; the lines within the boxes and whiskers denote the median and outer limits, respectively. (**C**) Kaplan-Meier analysis of overall survival for patients with ovarian cancer stratified by low miR-572 (≤ median, *n* = 54) and high miR-572 (> median, *n* = 54) expression. (**D**) Real-time PCR analysis of miR-572 expression in 12 freshly-isolated ovarian cancer tissue specimens and two normal ovarian tissue specimens. (**E**) Real-time PCR analysis of miR-572 expression in 13 ovarian cancer cell lines and a normal ovarian epithelial cell line (HOSEpiC). Transcript levels were normalized to *U6* expression. Experiments were repeated at least three times with similar results; values are mean ± SD; * *P* < 0.05.

By analyzing a published microarray-based high-throughput assessment (NCBI/E-MTAB-1067), we found that miR-572 was upregulated significantly (*P* < 0.0001) in human ovarian cancer tissues compared to normal ovarian tissues (Figure 1A). Expression of miR-572 was further examined in 108 archived clinical ovarian cancer specimens. As shown in Figure 1B, miR-572 was expressed at low levels in stage I and II tumors, markedly increased in stage III tumors and was further elevated in stage IV ovarian cancer. The *Chi*-squared test revealed that the levels of miR-572 strongly correlated with FIGO stage (*P* < 0.05; Figure 1B, Supplemental Table 2). Kaplan-Meier analysis and the log-rank test indicated that a high level of miR-572 expression was associated with significantly shorter overall survival (*P* < 0.001; Figure 1C, Supplemental Table 2). This data suggests a possible link between high-level miR-572 expression and the progression of human ovarian cancer, and highlights miR-572 may have potential value as a prognostic biomarker in ovarian cancer.

Real-time PCR analysis revealed that miR-572 was significantly overexpressed in 12 freshly-collected ovarian cancer samples compared to two normal ovarian tissues (Figure 1D). In agreement with these observations, upregulation of miR-572 was confirmed in 13 ovarian cancer cell lines compared with a control normal ovarian epithelial cell line (HOSEpiC; Figure 1E). Collectively, these results strongly indicate that miR-572 is upregulated in ovarian cancer.

### Overexpression of miR-572 promotes proliferation and cell cycle progression in ovarian cancer cells

**Figure 2:**
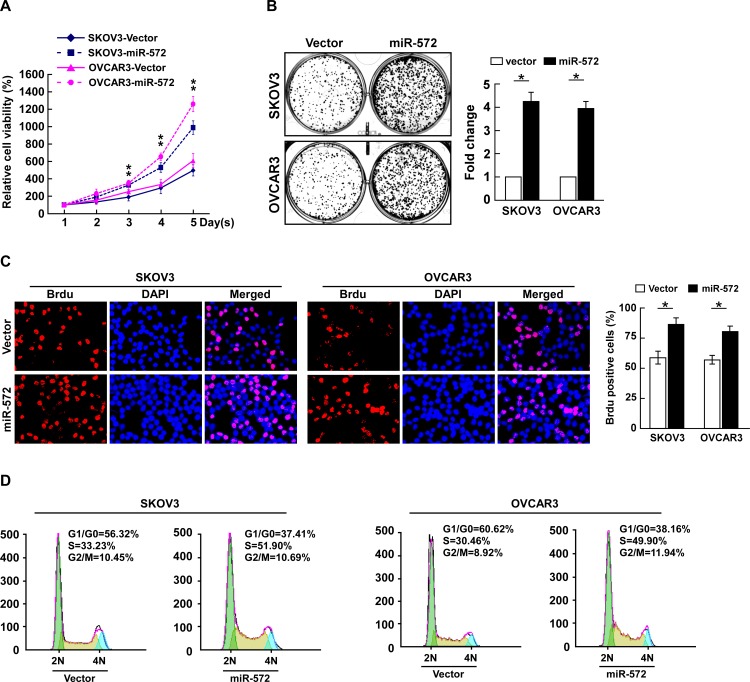
miR-572 promotes cell proliferation and cell-cycle progression in ovarian cancer cells (**A**) Effects of ectopic overexpression of miR-572 on the proliferation of the indicated ovarian cancer cell lines, as analyzed by the MTT assay. (**B**) Representative micrographs (left) and quantification (right) of colonies formed by the indicated ovarian cancer cell lines at 10 days after inoculation in the colony formation assay. (**C**) Representative micrographs (left) and quantification (right) of BrdUrd incorporating-cells for the indicated ovarian cancer cells. (**D**) Flow cytometric analysis of the effect of miR-572 overexpression on cell cycle progression for the indicated ovarian cancer cells. Experiments were repeated at least three times with similar results; values are mean ± SD; * *P* < 0.05.

To investigate the biological function of miR-572 in the development and progression of ovarian cancer, SKOV3 and OVCAR3 ovarian cancer cells stably expressing miR-572 were established (Supplemental Figure 1). The MTT assay demonstrated that ectopic overexpression of miR-572 significantly increased the growth rate of both SKOV3 and OVCAR3 cells (Figure 2A). The colony formation assay revealed that ectopic overexpression of miR-572 markedly enhanced the growth ability of both SKOV3 and OVCAR3 cells, as indicated by increased colony numbers and sizes (Figure 2B). The level of DNA synthesis, as examined using the BrdUrd incorporation assay, was significantly elevated in miR-572 transduced cells compared to vector control cells (Figure 2C). Furthermore, cell cycle analysis showed ectopic overexpression of miR-572 significantly increased the percentage of cells in the S phase and decreased the percentage of cells in the G1/G0 peak (Figure 2D). Collectively, these results demonstrate that miR-572 functions to enhance proliferation and cell cycle progression in ovarian cancer cells.

### Inhibition of miR-572 attenuates proliferation and cell cycle progression in ovarian cancer cells

**Figure 3:**
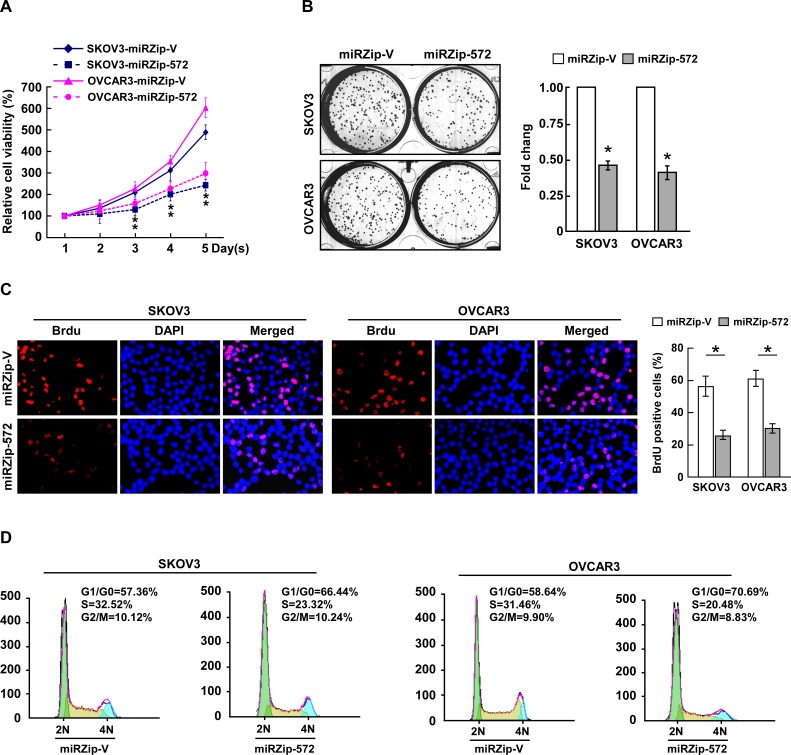
Inhibition of miR-572 reduces cell proliferation and cell-cycle progression in ovarian cancer cells (**A**) Effects of ectopic miR-572 on the proliferation of the indicated ovarian cancer cell lines, as analyzed by the MTT assay. (**B**) Representative micrographs (left) and quantification (right) of colonies formed by indicated ovarian cancer cell lines at 10 days after inoculation in the colony formation assay. (**C**) Representative micrographs (left) and quantification (right) of BrdUrd incorporating-cells for the indicated ovarian cancer cells. (**D**) Flow cytometric analysis of the effects of miR-572 overexpression on cell cycle progression for the indicated ovarian cancer cells. Experiments were repeated at least three times with similar results; values are mean ± SD; * *P* < 0.05.

Next, loss-of-function studies using a miR-572 inhibitor (miRZip-572) were performed to confirm the biological function of miR-572 in ovarian cancer progression. As shown in Figure 3A and 3B, suppression of miR-572 via transfection of the miR-572 inhibitor significantly decreased the growth rate of both ovarian cancer cell lines compared the respective negative control (miRZip-Vector) cells. The level of DNA synthesis was also significantly suppressed in miR-572-inhibited cells compared to control cells (Figure 3C). In addition, flow cytometry revealed a significant increase in the percentage of cells in the G1/G0 phase and decrease in the percentage of cells in the S phase in miR-572 inhibited cells (Figure 3D). These results suggest that downregulation of miR-572 inhibits proliferation, reduces tumorigenicity and prevents cell cycle progression in ovarian cancer cells.

### MicroRNA-572 suppresses the tumorigenicity of ovarian cancer cells both *in vitro* and *in vivo*

**Figure 4:**
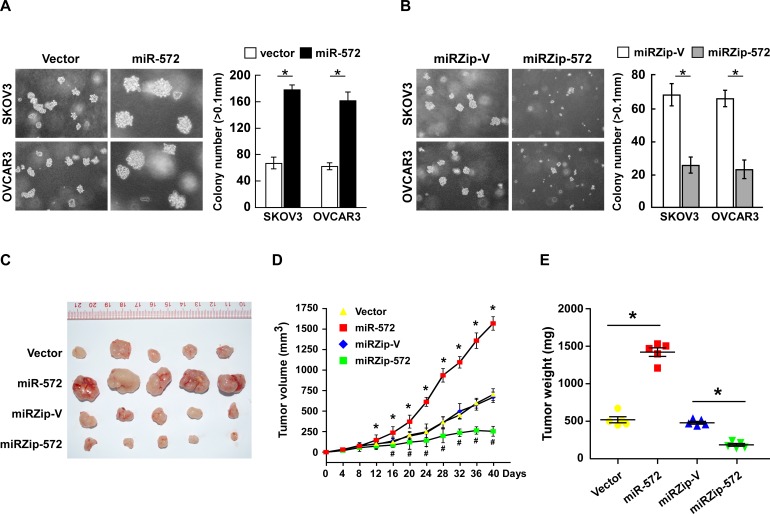
MiR-572 suppresses tumorigenicity of ovarian cancer cell both *in vitro* and *in vivo* (**A**) and (**B**) Representative micrographs (left) and quantification (right) of colony formation *in vitro* in the anchorage-independent growth assay. Colonies > 0.1 mm were scored. (**C**) Representative images of tumors formed by the indicated cells in the tumor xenograft model. (**D**) Average tumor volumes (mm^3^) for the indicated cells after inoculation. (**E**) Weight of the tumors formed by the indicated cells. * or # *P* < 0.05.

The anchorage-independent growth assay was performed to examine the effects of miR-572 on the tumorigenicity of ovarian cancer cells. Ovarian cancer cells stably expressing miR-572 formed higher numbers and larger colonies than the control cells, while inhibition of miR-572 led to the formation of fewer and smaller colonies (Figure 4A and 4B).

The biological effect of miR-572 on ovarian cancer progression was further examined using an *in vivo* tumor model. The miR-572-transduced ovarian cancer cells and miR-572-silenced cells, or the corresponding control cells, were subcutaneously injected into the dorsal flank of nude mice (Supplemental Figure 2). As shown in Figure 4C-4E, the tumors formed by miR-572- transduced ovarian cancer cells were larger, in both size and weight, than the corresponding control tumors, whereas the tumors formed by miR-572- silenced ovarian cancer cells were smaller in size and weight than the corresponding control tumors. These data indicate that miR-572 plays a pivotal role in ovarian cancer progression *in vivo*.

### MicroRNA-572 directly suppresses SOCS1 and p21 in ovarian cancer cells

**Figure 5:**
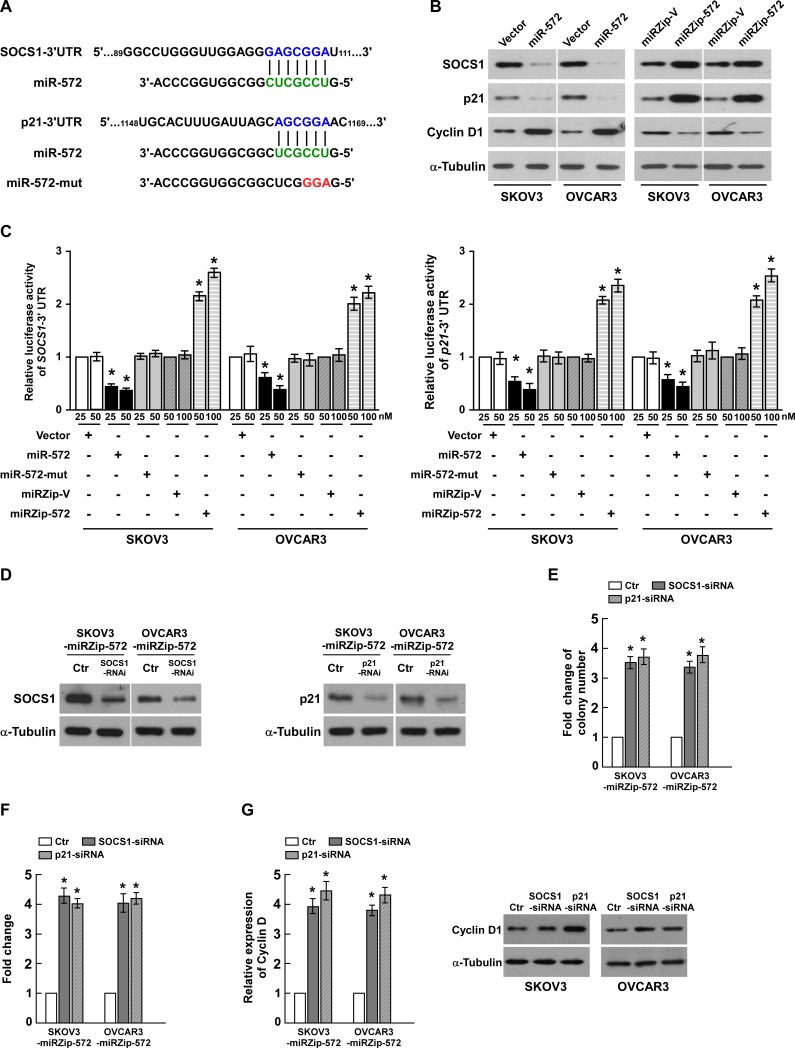
SOCS1 and p21 are essential for miR-572-mediated proliferation in ovarian cancer (**A**) Putative target sites for miR-572 in the 3` UTRs of *SOCS1* and *p21* and the sequence of miR-572 mutant (miR-572-mut). (**B**) Western blotting analysis of the protein levels of SOCS1, p21 and Cyclin D1 in the indicated cells. (**C**) Luciferase assays of the indicated cells co-transfected with the pGL3-*SOCS1* or *p21* reporter genes and miR-572, miR-572 inhibitor, miR-572 mutant (miR-572-mut) or the respective controls. (**D**) Western blotting analysis of the protein levels of SOCS1 and p21 in the indicated cells transfected with specific siRNAs, respectively. (**E**) Quantification of colonies formed by the indicated ovarian cancer cell lines in the colony formation assay. (**F**) Quantification of colonies formed by the indicated ovarian cancer cell lines in the anchorage-independent growth ability assay. (**G**) Real-time PCR analysis of Cyclin D1 mRNA expression and western blotting analysis of Cyclin D1 protein expression. Experiments were repeated at least three times with similar results; values are mean ± SD; * *P* < 0.05.

In an attempt to identify the mRNA targets of miR-572, we performed bioinformatic analysis using a publicly available algorithm (TargetScan 6.2). As shown in Figure 5A, *SOCS1* and *p21*, which are critical attenuators of cell proliferation and cell-cycle progression, were identified as potential targets of miR-572. Western blotting analysis showed that ectopic expression of miR-572 dramatically decreased, whereas inhibition of miR-572 increased, the protein expression levels of SOCS1 and p21 in both SKOV3 and OVCAR3 ovarian cancer cells (Figure 5B). Meanwhile, the expression of CyclinD1 was increased by ectopic expression miR-572, decreased by miR-572 inhibition (Figure 5B). Luciferase reporter plasmids containing regions of the 3` UTR of *SOCS1* or *p21* were constructed and cotransfected into ovarian cancer cells with miR-572, miR-572 inhibitor or the corresponding negative controls. As shown in Figure 5C, miR-572 significantly reduced the luciferase activity of the *SOCS1* and *p21* reporter genes, whereas transfection of the miR-572 inhibitor upregulated the luciferase activity of the reporter genes. However, transfection of miR-572-mut (miR-572 mutant) had no significant effect on the luciferase activity of the reporter genes (Figure 5C); and miR-572 also had no effect on the luciferase activity of the mutant reporter genes (Supplemental Figure 3). Taken together, these results confirm that *SOCS1* and *p21* are direct targets of miR-572.

### Suppression of SOCS1 or p21 are required for miR-572-induced cell proliferation and tumorigenesis in ovarian cancer

To evaluate the effects of SOCS1 and p21 on miR-572-induced ovarian cancer progression, we suppressed the expression of endogenous *SOCS1* or *p21* using specific siRNAs (Figure 5D). The colony formation assay indicated that silencing *SOCS1* or *p21* increased the proliferation of ovarian cells transfected with the miR-572 inhibitor (Figure 5E); similar results were observed in the anchorage-independent growth assay (Figure 5F). As shown in Figure 5G, silencing *SOCS1* or *p21* in miR-572-inhibitor transfected cells also increased the mRNA and protein expression of Cyclin D1, a well-characterized regulator of cell proliferation. These results suggest that silencing SOCS1 or p21 in miR-572-repressed cells reversed the negative effect of the miR-572 inhibitor on ovarian cancer cell proliferation and tumorigenesis.

### Clinical relevance of miR-572, SOCS1, p21 and Cyclin D1 in ovarian cancer

**Figure 6:**
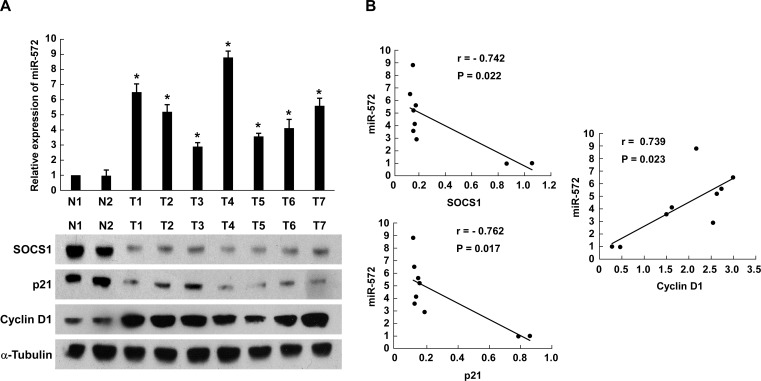
Expression of miR-572, SOCS1, p21 and Cyclin D1 in human ovarian cancer tissues (**A**) Real-time PCR analysis of miR-572 and Western blot analysis of SOCS1, p21 and Cyclin D1 expression in human ovarian cancer tissues. (**B**) Correlation between miR-572 expression and SOCS1, p21 and Cyclin D1 expression in ovarian cancer tissues. Experiments were repeated at least three times with similar results; values are mean ± SD; * *P* < 0.05.

**Figure 7:**
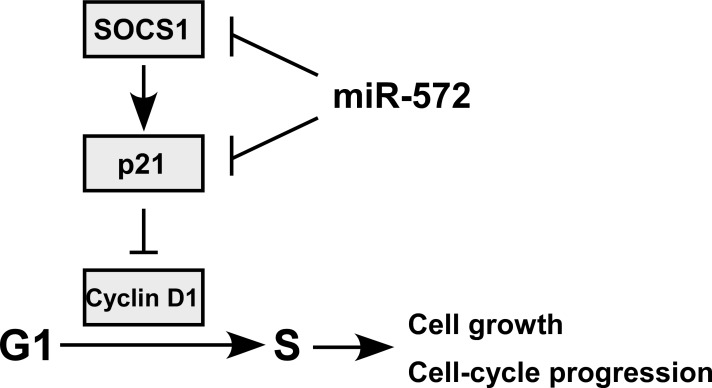
Proposed for model for the mechanism by which miR-572-mediated downregulation of SOCS1 and p21 promotes ovarian cancer cell proliferation

Finally, to examine whether miR-572-mediated suppression of SOCS1 and p21 in ovarian cancer is clinically relevant, seven freshly collected ovarian cancer samples and two normal ovarian tissues were obtained for further study. As shown in Figure 6A and 6B, the levels of miR-572 correlated with the protein expression levels of SOCS1 (*r* = −0.742, *P* = 0.022), p21 (*r* = −0.762, *P* = 0.017) and Cyclin D1 (*r* = 0.739, *P* = 0.023). These results suggest that miR-572 decreases the expression of SOCS1 and p21 and increases Cyclin D1 expression, consequently resulting in an aggressive phenotype and poorer prognosis in ovarian cancer (Figure 7).

## DISCUSSION

The discovery of effective diagnostic and prognostic biomarkers and therapeutic methods is urgently required for the diagnosis and treatment of ovarian cancer. Numerous studies have shown that miRNAs may represent valuable diagnostic and prognostic markers for cancer [32-35]. miR-572 is overexpressed in the serum of patients with nasopharyngeal carcinoma and renal cell carcinoma, and was used to construct a miRNA signature for patients prognosis [36, 37], and Li *et al*. found miR-572 was significantly overexpressed in peripheral T cell lymphoma, not otherwise specified (PTCL-NOS) [38]. The expression of miR-572 in ovarian cancer has not previously been investigated. This study demonstrates that miR-572 is significantly upregulated in ovarian cancer. Additionally, the expression of miR-572 correlated with ovarian cancer progression and overall patient survival, indicating that upregulation of miR-572 may contribute to the development of ovarian cancer and may have potential as a diagnostic and prognostic biomarker for ovarian cancer.

In the present study, *SOCS1* and *p21* were both identified as direct targets of miR-572 and could be suppressed by overexpression of miR-572, which in turn increased the proliferation and cell cycle progression of ovarian cancer cells *in vitro* and promoted tumorigenesis in an *in vivo* model of ovarian cancer. These findings are in agreement with the known roles of SOCS1 and p21 in cancer. Induction of SOCS1 by activated signal transducers and activators of transcription (STATs) can negatively regulate activation of the Janus tyrosine kinase (JAK)/STAT pathway. SOCS1 is upregulated when a cytokine binds to its cognate receptor, and in turn SOCS1 inhibits JAK activity by binding to its catalytic domain and recruiting the ubiquitin-transferase complex to target JAK for proteasomal degradation, leading to inactivation of JAK-related signaling and inhibition of tumor cell growth [39]. SOCS1 also controls STAT and toll-like receptor (TLR) signaling to exert a tumor suppressive function. By regulating this negative feedback mechanism, SOCS1 can influence the transduction of proliferative signals and affect the survival, differentiation and transformation of T cells or tumor cells [14, 40, 41]. It has been reported that SOCS1 inhibits cell growth, induces senescence and modulates cell cycle arrest resulting in G1 arrest in T cells, fibroblasts or tumor cells, and these effects were associated with upregulation of p21 [19].

The potent CDKI p21^CIP1/WAF1^ binds and inhibits the CDK2, CDK1 and CDK4/6 complexes, and functions as a negative regulator of cell proliferation and cell-cycle progression [16, 42]. For example, p21 inhibits cell proliferation by binding to and inhibiting Cyclin E/CDK2 kinase activity, leading to growth arrest at specific stages in the cell cycle [43]. p53-induced upregulation of p21 in response to DNA damage induces cell cycle blockade in G1, followed by DNA repair or induction of apoptosis [44]. In addition, p21 binds to proliferating cell nuclear antigen (PCNA) and interferes with PCNA-dependent DNA polymerase activity, thereby inhibiting DNA replication and modulating various PCNA-dependent DNA repair processes [45, 46]. Besides, p21 regulates gene transcription by directly associating with the promoter region of individual genes or by binding to specific transcription factors to modulate their activity. By directly controlling the activity of various tumor suppressors or oncogenes, p21 acts as an oncogene or tumor suppressor in different tumor types [47-49].

The evidence discussed above indicates that downregulation of SOCS1 and p21 may play essential roles in miR-572-induced tumor progression in ovarian cancer. However, the detailed regulatory network for miR-572, SOCS1 and p21, and the related signaling pathways are likely to be complicated and need further investigation. For example, it is already known that p21 is regulated by two different pathways: a p53-dependent pathway by which DNA damage leads to activation of p53 and upregulation of p21, and a p53-independent manner via which cellular growth factors, such as platelet-derived growth factor, fibroblast growth factor and epidermal growth factor induce p21 in p53-deficient quiescent cells [44]. The detailed regulatory mechanism of these factors has been under our further investigation.

Due to the ability of SOCS1 to inhibit various signaling pathways in tumor cells, the decreased expression of SOCS1 in cancer may represent a diagnostic and prognostic biomarker of clinical outcome in patients with myeloma, breast cancer, hepatocellular carcinoma, melanoma, or neuroendocrine tumor, suggesting SOCS1 may be a potential target for antitumor therapy [14, 15, 50-52]. However, it has been reported that assessment of the expression of p21 and p53 or other genes may be more useful as a marker of tumor progression and prognosis in tongue squamous cell carcinomas and oral squamous cell carcinomas [18, 44], and overexpression of p21 alone appeared to be insufficient to suppress tumor progression [53]. Our study demonstrates that miR-572, which is overexpressed in human ovarian cancer, can target and suppress both SOCS1 and p21, leading to ovarian cancer progression. Therefore, our results indicate the potential value of miR-572 in ovarian cancer development and miR-572 could be considered as useful biomarker for ovarian cancer diagnosis and prognosis. In addition, it was found that SOCS1 binds to p65 in the nucleus and operates as an E3 ligase that leads to polyubiquitination mediated proteasomal degradation of p65, resulting in suppression of p65 signaling and the expression of NF-κB signaling-regulated genes [54]. Based on this study, whether miR-572 performs its function via NF-κB signaling in ovarian cancer needs to be further investigated.

Besides, consistent with our results, miR-572 was found upregulated in various malignancies [37, 38]. However, miR-572 was downregulated in gastric cancer cell lines compared to normal gastric mucosa [55]. Zhu *et al*. found significant downregulation of miR-572 in chronic lymphocytic leukemia [56]. Moreover, miR-572 was also reported downregulation in basal cell carcinoma [57]. These results suggest that the same miRNA can exert distinct biological activities under different cellular contexts.

In conclusion, miR-572 is overexpressed in ovarian cancer and upregulation of miR-572 promoted ovarian cancer cell proliferation, cell-cycle progression and tumorigenicity both *in vitro* and *in vivo*, while inhibition of miR-572 lead to the opposite effects. Moreover, the function of miR-572 in ovarian cancer may be exerted via downregulation of the target genes *SOCS1* and *p21*, which play an important role in the function of miR-572 in ovarian cancer. Therefore, this study demonstrates that miR-572 may play an important role in ovarian cancer progression and may represent a potential therapeutic target for ovarian cancer therapy.

## MATERIALS AND METHODS

### Cell culture and treatments

Human ovarian epithelial cell (HOSEpiC) were purchased from Sciencell Research Labs (Carlsbad, CA, USA) and maintained according to the manufacturer's instruction. The ovarian cancer cell lines OVCAR3, SKOV3, TOV-21G, TOV-112D, A2780, CAOV3, CAOV4, OV56, OV90, COV362, COV434, COV504 and COV644 were maintained in RPMI 1640 (Invitrogen, Carlsbad, CA, USA) supplemented with 10% FBS (Invitrogen), at 37°C in a 5% CO_2_ atmosphere in a humidified incubator.

### Tissue specimens and patient information

The 108 paraffin-embedded, archived ovarian cancer samples and freshly collected ovarian cancer tissue specimens used in this study were histopathologically and clinically diagnosed at the Sun Yat-sen University Cancer Center between 2008 and 2011. The disease stages of all the patients were classified according to the International Federation of Gynecology and Obstetrics (FIGO) guidelines for clinical staging. The clinicopathological characteristics of the samples are summarized in Supplemental Table 1. Normal ovarian tissues were obtained from individuals who underwent wedge biopsy of the ovaries and confirmed to be free of any pre-existing pathologically detectable conditions. All samples were collected and analyzed with prior written informed consent from the patients. Prior donors' consents and approvals from the Institutional Research Ethics Committee were obtained.

### Generation of stably engineered cell lines

pMSCV-miR-572 was generated by cloning the genomic pre-miR-572 gene with about 500-bp on each flanking side (primers used: forward, 5′-GCCAGATCTCTGAGGAAAGCAGGAGGAGG -3′; reverse, 5′- GCCGAATTCTCGG CACAAATCTTCAGAGC -3′) into the retroviral transfer plasmid pMSCV-puro (Clontech Laboratories Inc., Mountain View, CA, USA). pMSCV-miR-572 was then cotransfected with the pIK packaging plasmid into 293FT cells, using the standard calcium phosphate transfection method [30]. Thirty-six hours after transfection, the supernatants were collected and then incubated with ovarian cancer cells to be infected for 24 hours in the presence of polybrene (2.5 μg/ml, Sigma, Saint Louis, MO, USA). Puromycin (1.5 μg/ml, Sigma) was used to select stably transduced cells two weeks after infection.

### RNA extraction and real-time quantitative PCR

Total cellular RNA was extracted using Trizol reagent (Invitrogen), according to the manufacturer's instruction. cDNAs were synthesized and real-time PCR was performed using the GoTaq^®^ 2-Step RT-qPCR System (Promega, Madison, WI, USA). SYBR Green I (Invitrogen) was used to quantify PCR amplification and the qRT-PCR was performed and analyzed using a 7500 Fast Real-Time Sequence detection system (Applied Biosystems, Foster City, CA, USA). miRNA quantification was determined by using Bulge-loop^TM^ miRNA qRT-PCR Primer Set (one RT primer and a pair of qPCR primers for each set) specific for miR-572, designed by RiboBio (RiboBio Co. Ltd, Guangzhou, Guangdong, China). The expression of the miRNA was defined based on Ct, and relative expression levels were calculated as 2^−[(Ct of *miR-572*) -(Ct of *U6*)]^ after normalization with reference to the expression of small nuclear RNA U6. Expression levels of genes were normalized to that of the housekeeping gene GAPDH as the control and calculated as 2^−[(Ct of *GENES*) - (Ct of *GAPDH*)]^ (Ct, represents the threshold cycle for each transcript). The following primers were used: *Cyclin D1* forward, 5′-AACTACCTGGACCGCTTCCT-3′, and reverse, 5′-CCACTTGAGCTTGTTCACCA-3′. *GAPDH* forward: 5′- GACTCAT GACCACAGTCCATGC -3′, reverse: 3′- AGAGGCAGGGATGATGTTCTG -5′.

### Oligonucleotides, siRNA and transfection

The miR-572 anti-sense (miRZip-572) plasmid used as miR-572 inhibitor, and the vector control (miRZip-Vector) were purchased from System Biosciences (San Francisco, CA) and used according to previous report [31]. For depletion of *SOCS1-*, and *p21-*siRNAs were synthesized and purified by RiboBio. Transfection of oligonucleotides and siRNAs were performed using the Lipofectamine 2000 reagent (Invitrogen), according to the manufacturer's instruction.

### Western blotting analysis

Total protein was extracted from whole cells and 20 μg of isolated protein was separated by SDS-PAGE and electroblotted onto a PVDF membrane (Bio-Rad Laboratories, Hercules, CA, USA). The membranes were then probed with antibodies: anti-SOCS1, anti-p21, anti-Cyclin D1 (Abcam, Cambridge, MA, USA). The membranes were stripped and reblotted with an anti-α-tubulin monoclonal antibody (Sigma) as a loading control.

### Colony formation assay

Cells were plated on 6-well plate (1000 cells per dish) and cultured for 10 days. The colonies were stained with 0.1% crystal violet for 5 min after fixation with 10% formaldehyde for 15 min. Viable colonies that contained more than 50 cells were counted. The experiment was performed for three independently times for each cell line.

### Anchorage-independent growth ability assay

Cells (1×10^3^) were trypsinized and suspended in 2 ml complete medium plus 0.33% agar (Invitrogen) and plated in 6-well plate on top of a bottom agar layer (0.66% complete medium agar). After two-week days incubation, colony sizes were measured with an ocular micrometer and colonies greater than 0.1 mm in diameter were counted. All experiments were performed in triplicate.

### Bromodeoxyuridine labeling and immunofluorescence

Cells grown on coverslips (Fisher, Pittsburgh, PA, USA) were incubated with bromodeoxyuridine (BrdUrd) for 1 h and stained with anti-BrdUrd antibody (Sigma) according to the manufacturer's instruction. Gray level images were acquired under a laser scanning microscope (Axioskop 2 plus, Carl Zeiss Co. Ltd., Jena, Germany). All experiments were performed in triplicate.

### Flow cytometry analysis

All cells in a culture dish were harvested by trypsinization, washed in ice-cold PBS, and fixed in 80% ice-cold ethanol in PBS. Before staining, the cells were spun down in a cooled centrifuge and resuspended in the cold PBS. Bovine pancreatic RNAase (Sigma) was added at a final concentration of 2 μg/ml, and cells were incubated at 37°C for 30 min, followed by incubation in 20 μg/ml of propidium iodide (Sigma) for 20 min at room temperature. Fifty thousand cells were analyzed flow cytometrically. All experiments were performed in triplicate.

### Luciferase assay

Cells were seeded in triplicate in 24-well plate and allowed to settle for about 12h. One hundred nanograms of pGL3- *SOCS1*, or - *p21*-luciferase plasmid was co-transfected into ovarian cancer cells with TK-Renilla plasmid as control signals using the Lipofectamine 2000 reagent, according to the manufacturer's instruction. Luciferase and control signals were measured at 48h after transfection using the Dual Luciferase Reporter Assay Kit (Promega, Madison, WI, USA), according to a protocol provided by the manufacturer. Three independent experiments were performed and the data were presented as the mean ± SD.

### Xenografted tumor model

BALB/c-nude mice (female, 4-5 weeks of age, 18-20g) were purchased from the Center of Experimental Animal of Guangzhou University of Chinese Medicine. All experimental procedures were approved by the Institutional Animal Care and Use Committee of Sun Yat-sen University. The BALB/c nude mice were randomly divided into two groups. One group of mice was inoculated subcutaneously with SKOV3/Vector cells (5×10^6^) in the left dorsal flank and with SKOV3/miR-572 cells (5×10^6^) in the right dorsal flank per mouse. Another group was inoculated subcutaneously with SKOV3/miRZip-Vector cells (5×10^6^) in the left dorsal flank and with SKOV3/miRZip-572 cells (5×10^6^) in the right dorsal flank. Tumors were examined once every 4 days; length, width, and thickness were measured with callipers, and tumor volumes were calculated. Tumor volume was calculated using the equation (L*W^2^)/2. Forty days after tumor implantation, the mice were killed, the tumors were removed and weighed.

### Statistical analysis

Student's *t* test was used to evaluate the significant difference between two groups of data in all the pertinent experiments. The experimental data were represented from three biological independent replicates and as the mean ± SD. Bivariate correlations between study variables were calculated by Spearman's rank correlation coefficients. Survival curves were plotted by the Kaplan-Meier method and compared by the log-rank test. A *P* value < 0.05 (using a two-tailed paired *t* test) was considered statistically significant.

## SUPPLEMENTARY MATERIAL, FIGURES AND TABLES


