# Supplementary material for: Upregulation of miR-572 transcriptionally suppresses SOCS1 and p21 and contributes to human ovarian cancer progression
Source: Oncotarget. 2015 Mar 30;6(17):15180–93. doi: 10.18632/oncotarget.3737 (PMC4558144; doi:10.18632/oncotarget.3737)
Supplement: Supplementary file 1 [file oncotarget-06-15180-s001.pdf]

Upregulation of miR-572 transcriptionally suppresses SOCS1 and p21 and contributes to human ovarian cancer progression

Supplementary Material

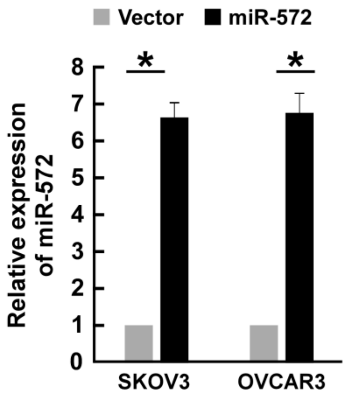

Supplemental Figure 1 : Real-time PCR analysis of miR-572 expression in ovarian cancer cell lines stably expressing miR-572 and vector control cells. Transcript levels were normalized to *U6* expression. Experiments were repeated at least three times with similar results; values are mean ± SD; * $P < 0.05$.

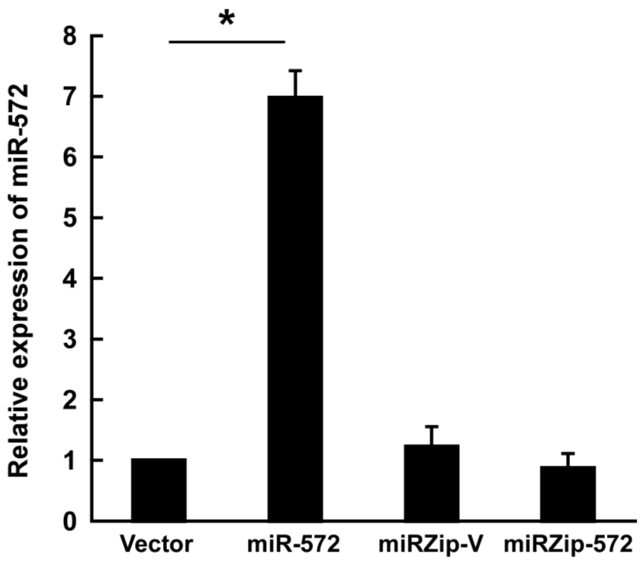

Supplemental Figure 2: Real-time PCR analysis of miR-572 expression in the indicated ovarian cancer cell lines. Transcript levels were normalized to *U6* expression. Experiments were repeated at least three times with similar results; values are mean ± SD; * $P < 0.05$.

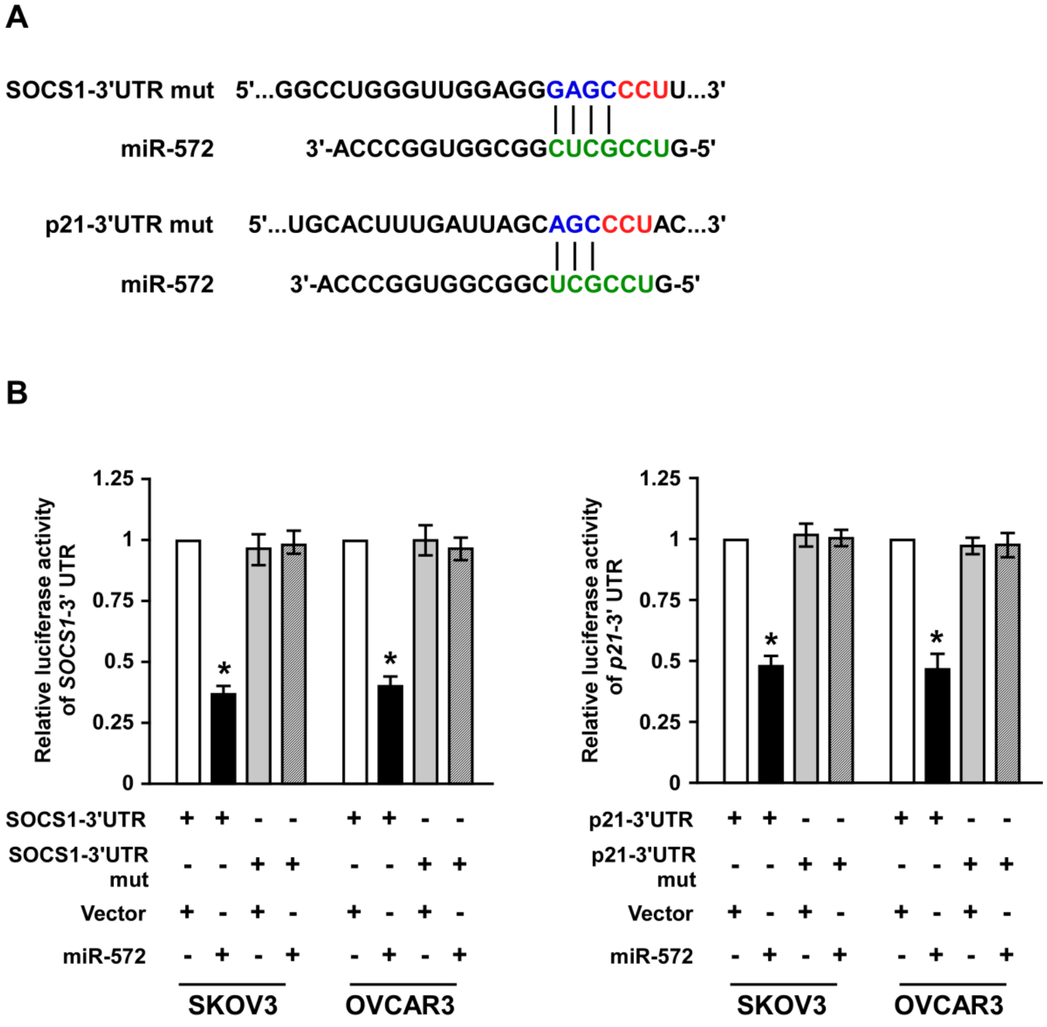

Supplemental Figure 3: SOCS1 and p21 are essential for miR-572-mediated proliferation in ovarian cancer. (A) The sequences of 3' UTRs of *SOCS1* and *p21* mutant. **(B)** Luciferase assays of the indicated cells co-transfected with the pGL3-*SOCS1* or -*p21* mutant reporter genes and miR-572 or the vector control.

Supplementary Tables

Supplementary Table 1: Clinicopathological characteristics of studied patients and expression of miR-572 in ovarian cancer

Factor	No.	(%)
**Age (years)**		
≤45	40	37.0
>45	68	63.0
**Clinical stage**		
I	32	29.6
II	16	14.8
III	43	39.8
IV	17	15.7
**Vital status**		
Alive	76	70.4
Dead	32	29.6
**Expression of miR-572**		
Low expression	54	50
High expression	54	50

Supplementary Table 2: Correlation between the clinicopathological features and expression of miR-572 in ovarian cancer

**Patient characteristics**		**miR-572 expression**		***P*-value**
		**Low**	**High**	
**Age (years)**	≤45	28	12	0.001
	>45	26	42	
**Clinical stage**	I	32	0	<0.001
	II	13	3	
	III	8	35	
	IV	1	16	
**Survival time** (Median=35 month)	≤35	8	48	<0.001
	>35	46	6	
**Vital status**	Alive	46	30	0.001
	Dead	8	24	
